# Acid Hydrolysis and Molecular Density of Phytoglycogen and Liver Glycogen Helps Understand the Bonding in Glycogen α (Composite) Particles

**DOI:** 10.1371/journal.pone.0121337

**Published:** 2015-03-23

**Authors:** Prudence O. Powell, Mitchell A. Sullivan, Joshua J. Sheehy, Benjamin L. Schulz, Frederick J. Warren, Robert G. Gilbert

**Affiliations:** 1 Tongji School of Pharmacy, Huazhong University of Science and Technology, Wuhan, Hubei, China; 2 Centre for Nutrition and Food Sciences, Queensland Alliance for Agriculture and Food Innovation, The University of Queensland, Brisbane, QLD, Australia; 3 School of Chemistry and Molecular Biosciences, Faculty of Science, The University of Queensland, Brisbane, QLD, Australia; Faculty of Biology, SPAIN

## Abstract

Phytoglycogen (from certain mutant plants) and animal glycogen are highly branched glucose polymers with similarities in structural features and molecular size range. Both appear to form composite α particles from smaller β particles. The molecular size distribution of liver glycogen is bimodal, with distinct α and β components, while that of phytoglycogen is monomodal. This study aims to enhance our understanding of the nature of the link between liver-glycogen β particles resulting in the formation of large α particles. It examines the time evolution of the size distribution of these molecules during acid hydrolysis, and the size dependence of the molecular density of both glucans. The monomodal distribution of phytoglycogen decreases uniformly in time with hydrolysis, while with glycogen, the large particles degrade significantly more quickly. The size dependence of the molecular density shows qualitatively different shapes for these two types of molecules. The data, combined with a quantitative model for the evolution of the distribution during degradation, suggest that the bonding between β into α particles is different between phytoglycogen and liver glycogen, with the formation of a glycosidic linkage for phytoglycogen and a covalent or strong non-covalent linkage, most probably involving a protein, for glycogen as most likely. This finding is of importance for diabetes, where α-particle structure is impaired.

## Introduction

Phytoglycogen is a highly branched glucan found in certain mutant plants that arises as a consequence of a variety of mutations that result in disruptions in the gene encoding the isoamylase debranching enzyme. An example of one of these mutations is the *Sugary-1* mutant maize line [1–4] Glycogen is a structurally similar polymer involved in glucose regulation synthesized by animals [5]. Both function as glucose storage polymers, and are made of α-(1→4)-linked glucose units forming linear chains that are joined together via α-(1→6) glycosidic linkages as branch points. Glycogen is also associated with small but significant amounts of protein [6–8]. The size distribution of liver glycogen, ranging from ~20 to 300 nm in diameter, shows two components [9]. The smaller comprises what appear in transmission electron microscopy (TEM) to be homogenous entities termed β particles (~ 20 nm in size). These particles can join together, by a mechanism which is presently unclear, to form larger α particles (50–300 nm) which have a composite, cauliflower-like, appearance. TEM images of phytoglycogen also show composite particles [10], and phytoglycogen has a monomodal size distribution of particles with a similar size range to that of glycogen [9, 11]. Here we use the terms β and α particles to denote small particles with apparently homogeneous morphology, and large particles with apparently composite morphology, respectively, always recognizing that β particles in one glycan may be structurally different from those in the other, and similarly for α particles.

For liver glycogen, the formation of large α particles correlates with normal blood glucose regulation in mice [12, 13]. This finding is based on the discovery that *db/db* mice (which serve as a model for type II diabetes) display altered α-particle formation [13]. It is not known if the lack of large α particles observed in the size distributions of liver glycogen dissolved in dimethyl sulfoxide from *db/db* mice contributes to the development of type II diabetes, is a result of the disease, or if it is simply a feature of the *db/db* mouse. Nor is it known how the smaller β particles are bound to form large α particles in liver glycogen. Possible binding mechanisms previously explored include aggregation through hydrogen bonding/hydrophobic effects [14], protein disulfide bonds [15, 16] and glycosidic linkages [14]. In principle, proteomics could provide a direct answer to this problem by finding differences in the bound proteins in α and β particles, but this is not a simple task. Removing the abundant unbound proteins from a liver sample is a major challenge and even the best current work on this can be seen not to overcome this problem [8]. It is for this reason that we have undertaken the present work which seeks information on this binding using indirect experimental evidence, especially the time evolution of the size distribution during acid hydrolysis and the size distribution of the molecular density.

Recent work looking at changes in glycogen particle size distributions during acid hydrolysis has determined that the bonding between components in liver glycogen is unlikely to be hydrogen or glycosidic bonds, with the most likely candidate being a linkage where small particles formed by glycosidic linkages are joined by a covalent or non-covalent bond involving a protein, which we have termed a protein “glue” [14].

The objective of this paper is to obtain information on the binding of small β particles into large composite α particles in liver glycogen. This binding is impaired in diabetic liver glycogen, and so the knowledge gained could prove useful in the management of diabetes. In this paper, this is done by comparing certain properties of glycogen (synthesized in animals) and phytoglycogen (synthesized in plants); these properties are the evolution of molecular size distribution during acid hydrolysis and the size dependence of molecular density. Glycogen and phytoglycogen are structurally very similar. Further, the branching enzymes involved in the biosynthesis of both molecules have similarities, e.g. in the conserved sequences close to the binding sites. This makes phytoglycogen a good control to explore the binding in liver glycogen, especially because phytoglycogen biosynthesis and regulation involves fewer proteins than does glycogen synthesis in the liver.

Acid hydrolysis is expected to occur uniformly throughout the particles, while enzymatic degradation is probably confined to the exterior of the glycogen particles. Understanding any quantitative and qualitative differences in the time evolution of the size distributions of phytoglycogen and liver glycogen during acid hydrolysis would help to understand any structural differences. The reason for comparing these two particles is that a) liver glycogen has two distinct populations, which may be assigned as α and β particles, whereas phytoglycogen has only a single population which also contains some composite particles and covers a similar size range as the α and β particles in liver glycogen. Comparing these two distinct glucans can aid in finding any structural differences between the isolated β particles in glycogen, the individual β particles in glycogen α particles, and the individual β-like particles in phytoglycogen α-like particles, particularly as the α and β particle populations are extremely difficult to isolate from one another due to the overlap of the populations. Characterizing the fully branched structures of phytoglycogen and liver glycogen will aid in our understanding of the differences in their structures and could provide important clues to help us understand α-particle linkage. This may further our understanding of the role of these particles in type II diabetes.

## Materials and Methods

### Materials

Grain from *sugary-1* mutant maize plants was obtained from Prof. Ian D. Godwin (The University of Queensland, Brisbane, Australia) and ground to a fine powder in a cryo-mill (Freezer.Mill 6870, SPEC CertiPrep, Metuchen, NJ, USA; 1 min precooling followed by 5 min grinding), a technique that has been shown to minimize degradation of glucan polymers [17]. Phytoglycogen in the ground grain was extracted and purified as described in a previous study [11]; it is noted that the mature grain does not show diurnal cycling in starch/phytoglycogen content. Kernel flour (100 mg) was weighed into a centrifuge tube and incubated in a 2.5 mL aqueous solution of protease (2.5 units/mL; bacterial type XIV, Sigma-Aldrich, Castle Hill, NSW, Australia) and tricine buffer (pH 7.5, 250 mM) solution at 37°C for 30 min. An additional 2.5 mL of cold tricine buffer was added to the sample after incubation then centrifuged at 4000 g for 10 min. The supernatant was collected and the phytoglycogen precipitated using approximately four volumes of absolute ethanol. The solution was centrifuged at 4000 g for 10 min and the precipitate washed once more with ethanol. The pellet was re-suspended in deionized water before being lyophilized (freeze-dried; VirTis, Benchtop K).

Glycogen was extracted from pig livers and purified as described in a previous study [14]. The liver sample was collected from the central lobe of the liver, immediately frozen in dry ice and kept at -80°C for 6 weeks before glycogen analysis. The liver glycogen was homogenized in five volumes of cold (4°C) glycogen isolation buffer (50 mM Tris, pH 8, 150 mM NaCl, 2 mM EDTA, 50 mM NaF, 5 mM sodium pyrophosphate, and protease-inhibiting phenylmethansulfonylfonylfluoride (PMSF). Tris has been shown to be a potent inhibitor of glycosidase activity [18]. The sample was centrifuged at 6000 g for 10 min at 4°C with the resulting supernatant centrifuged further at 50000 g for 30 min at 4°C. The pellet was re-suspended in glycogen isolation buffer and layered over a stepwise sucrose gradient (25, 50 and 75% in glycogen isolation buffer) before being centrifuged at 300 000 g for 2 h at 4°C. The pellet containing the extracted glycogen was re-suspended in 80% ethanol and centrifuged at 4000 g for 10 min at 4°C before the supernatant was discarded. The ethanol washing step was repeated once more and the pellet was re-suspended in deionized water before being lyophilized (freeze-dried; VirTis, Benchtop K).

### Transmission Electron Microscopy (TEM)

TEM imaging was conducted as previously described [11]. A 400-mesh grid (ProSciTech, Formvar with heavy carbon coating) was glow-discharged before use. Droplets of approx. 0.01% phytoglycogen or liver glycogen dispersed in distilled water were placed on the grid for ~ 1.5 min before staining with 2% aqueous uranyl acetate. The particles were imaged on a JEOL 1010 TEM (Tokyo, Japan) operating at 100 kV at the UQ Centre for Microscopy and Microanalysis. Images were digitally recorded with a SIS Veleta CCD camera (Olympus, Münster, Germany) and AnalySiS image management software.

### Size Exclusion Chromatography (SEC)

SEC (also termed GPC) separates polymers based on their hydrodynamic volume (*V*_h_) or the corresponding hydrodynamic radius *R*h. The resulting information is presented here as the SEC weight distribution, *w*(log *R*_h_), which is the same as *w*(log *V*h) to within an arbitrary factor.

One limitation of our earlier work on acid hydrolysis was that the distributions were analyzed by DMSO/LiBr SEC. This system does not give clear separation of the peaks of α and β particle populations in liver glycogen. Recently it has been found that aqueous-based SEC systems are able to produce distributions with improved separation of these two particle populations [19]. SEC for liver glycogen and phytoglycogen here uses an aqueous eluent [9, 20]. An AF2000 SEC set-up (Postnova Analytics, Landsberg-Lech, Germany) with a flow rate of 0.3 mL/min was used to analyze the structures of branched phytoglycogen and liver glycogen. SUPREMA preColumn 30 and 3000 (PSS Germany) analytical columns were used, which provide good resolution for glycogen in aqueous SEC [9]. These columns were placed in a column oven at 80°C with 50 mM NH_4_NO_3_ containing 0.02% sodium azide as the eluent. A refractive index detector (RID; Shimadzu RID-10A, Shimadzu, Japan) was used.

Universal calibration curves were obtained using pullulan standards (PSS) with a molecular weight range of 342 to 2.35 × 10^6^. The Mark-Houwink parameters used were *K* = 1.0176 × 10^–3^ dL g^–1^ and *α* = 0.525, giving an *R*_h_ upper limit of calibration of ~44 nm (Prof. Katja Loos, University of Groningen, private communication). Elugrams were smoothed using a 5-point moving-point average.

A significant disadvantage of SEC is the occurrence of band-broadening, which can distort the shape of the distribution and mask fine features. For the present purposes, these problems do not affect the conclusions drawn from the data.

### Acid Hydrolysis

Previous examination of the effects of low pH on liver glycogen structure indicated that protein may be involved in the binding of small liver glycogen β particles to form α particles [14]. A modified method to that used previously [14] was employed here for acid hydrolysis of both phytoglycogen and liver glycogen.

Phytoglycogen and liver glycogen were dissolved in 0.1 M sodium acetate buffer (2 mg/mL; pH ~3.5) and heated to 80°C in a thermomixer for timed intervals between 10 min and 14 days. All samples were run in duplicate. The samples were removed from the thermomixer after the designated period of time and the phytoglycogen and liver glycogen precipitated with four volumes of ethanol. Samples were centrifuged for 10 min at 4000 g and the supernatant discarded. Samples were dissolved in aqueous (ammonium nitrate) eluent (50 mM NH_4_NO_3_ with 0.02% sodium azide) for SEC analysis. As an additional control, phytoglycogen and liver glycogen were dissolved in deionized water (2 mg/mL; performed in duplicate) and heated in the thermomixer at 80°C for 7 days. The samples were precipitated in four volumes of absolute ethanol then centrifuged (4000 g for 10 min). They were then dissolved in the aqueous eluent and characterized by SEC.

### Model for the Uniform Degradation of Glucans during Acid Hydrolysis over Time

Data interpretation was aided by the following mathematical model describing the time evolution of the size distribution for the ideal case where acid hydrolysis of (phyto)glycogen occurs uniformly and randomly throughout the molecule. This assumption is reasonable because of the mobility and small size of hydrogen ions. It is also assumed that the radius of gyration is approximately the SEC hydrodynamic radius, *R*g ≈ *R*h, which seems to be the case for amylopectin [21], whose structure in solution has many similarities to that of liver glycogen and phytoglycogen. It is further assumed that the “local dispersity” is unity: that number and weight averages of the molecular weight are the same for a given size, ${\bar{M}}_{n}\left(R_{\text{h}}\right)={\bar{M}}_{w}\left(R_{\text{h}}\right)$. This is probably a reasonable approximation because liver glycogen and phytoglycogen particles are large and randomly hyperbranched, and this relation is indicated by theory under these circumstances [22]; this monodispersity is also indicated by data from multiple-detection SEC [19]. For notational simplicity, we put *V*h = *V* and *R*h = *R*.

The SEC size distribution and number distribution are denoted *w*(log *R*) and *N(R*), respectively. Under the assumption of unit local dispersity [23], one has:

$$w\left(\text{log}R\right)={\left[M\left(R\right)\right]}^{2}\;N\left(R\right)$$ (1)

Here $M\left(R\right)={\bar{M}}_{\text{n}}\left(R\right)={\bar{M}}_{\text{w}}\left(R\right)$. Further, because of the hyperbranched nature of the polymer, if one assumes that the internal density inside a glycogen molecule is uniform, then *M*(*R*) ∝ *V* ∝ *R* ^3^.

By a straightforward change in the derivation of the time evolution of the particle size distribution of the growth of synthetic polymer colloids [24], one then has the following evolution equation for the number distribution under uniform degradation:

$$\frac{\partial N(R,t)}{\partial t}=\frac{\partial }{\partial V}(KN)$$ (2)

Here *K*(*V*) is the rate of degradation. The derivation of this result is given in S1 Text. It is assumed that *K*(*V*) is independent of the internal structure of the molecule but proportional to the amount of glucose monomer within it; one then has

K(V)=kV (3)

where the rate coefficient *k* is a constant. Eq. 2 is solved numerically using finite difference, as set out in S1 Text. As also shown in S1 Text, when one treats the data by comparing results in terms of dimensionless time τ = *kt*, there are no adjustable parameters in this model, and one then obtains the value of *k* by finding the value of τ giving the best fit between the calculated and observed size distributions for a given experimental time *t*.

**Fig 1:**
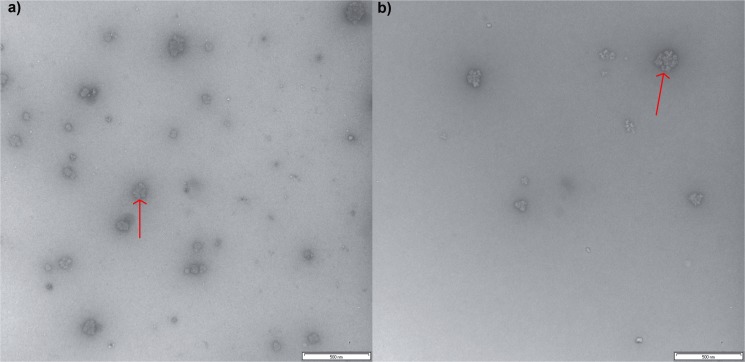
Aqueous SEC weight distributions of acid hydrolyzed glucans. Phytoglycogen (a) and liver glycogen (b) particle samples were taken over 14 days of acid hydrolysis. The following terms have been abbreviated: minute: min; hours: h; days: d. Curves have been normalized to equal areas.

Fitting was performed over each of the time intervals for which data were obtained. In each case, the initial distribution of *w*(log *R*h) was chosen as that at the start of that interval. The first value was taken to be that at 10 min. This is because of the short but significant time (10 min) required for sample preparation and equilibration meant that the original starting material without such preparation is probably different from what would be the true *t* = 0 material, if it were possible to extract a zero-time sample in a system that were infinitely fast to equilibrate. Additionally, the sample at the beginning of each experiment was not prepared in exactly the same way as further samples, in that there was no ethanol precipitation. This may have affected the subsequent distribution, as ethanol precipitation may preferentially select for larger sized particles. Indeed, as seen in Fig. 1, the distributions of the sample prior to hydrolysis are anomalous compared to those later in the hydrolysis. Due to this difference between the starting sample and 10 and 30 min time points, subsequent discussion of the acid hydrolysis results does not consider the starting distribution.

### Density Distributions of Phytoglycogen and Liver Glycogen

The molecular density at a given hydrodynamic radius is defined here as (weight-average molar mass)/(z-average volume of gyration), with ${\bar{M}}_{\text{w}}$ and *R*gz both being functions of hydrodynamic radius:

$$\rho (R_{h})=\frac{{\bar{M}}_{\text{w}}}{\frac{4}{3}\pi R_{gz}{}^{3}}$$ (4)

Some workers who have examined molecular density (e.g. Fernandez, Rojas and Nilsson [25]) have omitted the factor of 4/3 π, but the definition given here enables comparison with macroscopic densities: thus for example one expects the definition defined as above to be commensurate with that of water, ~ 1 g cm–3. The densities of phytoglycogen and liver glycogen were obtained using SEC with differential refractive index (DRI) and multi-angle laser light scattering (MALLS) detection to measure ${\bar{M}}_{\text{w}}$ and *R*gz. Three phytoglycogen samples were extracted from *su-1* maize grain (Phyto 1, Phyto 2 and Phyto 3; extracted as described in a previous study [11]), and liver glycogen from mice and pigs (Mouse 1, Mouse 2, Mouse 3 and Pig 1) were extracted using methods outlined previously [14]. Approximately 1–2 mg of each sample was dissolved in 500 μL of 50 mM NH_4_NO_3_ containing 0.02% sodium azide in an Eppendorf tube. Samples were placed in a thermomixer at 80°C at 350 rpm for 8 h, before being centrifuged at 4000 *g* for 10 min.

SEC was performed using an Agilent 1100 Series SEC system (Agilent Technologies, Santa Clara, USA) with a MALLS detector (Wyatt, Santa Barbara, CA, USA), and a flow rate of 0.3 mL/min. SUPREMA preColumn 1000 and 10000 (PSS Germany) analytical columns were used, which provide good resolution for glycogen in aqueous SEC [9]. These columns were placed in a column oven at 80°C with the aqueous eluent. Data were reduced to ${\bar{M}}_{\text{w}}$ (log *R*h) and *R*gz(log *R*h) using a Berry plot.

## Results

### TEM

**Fig 2:**
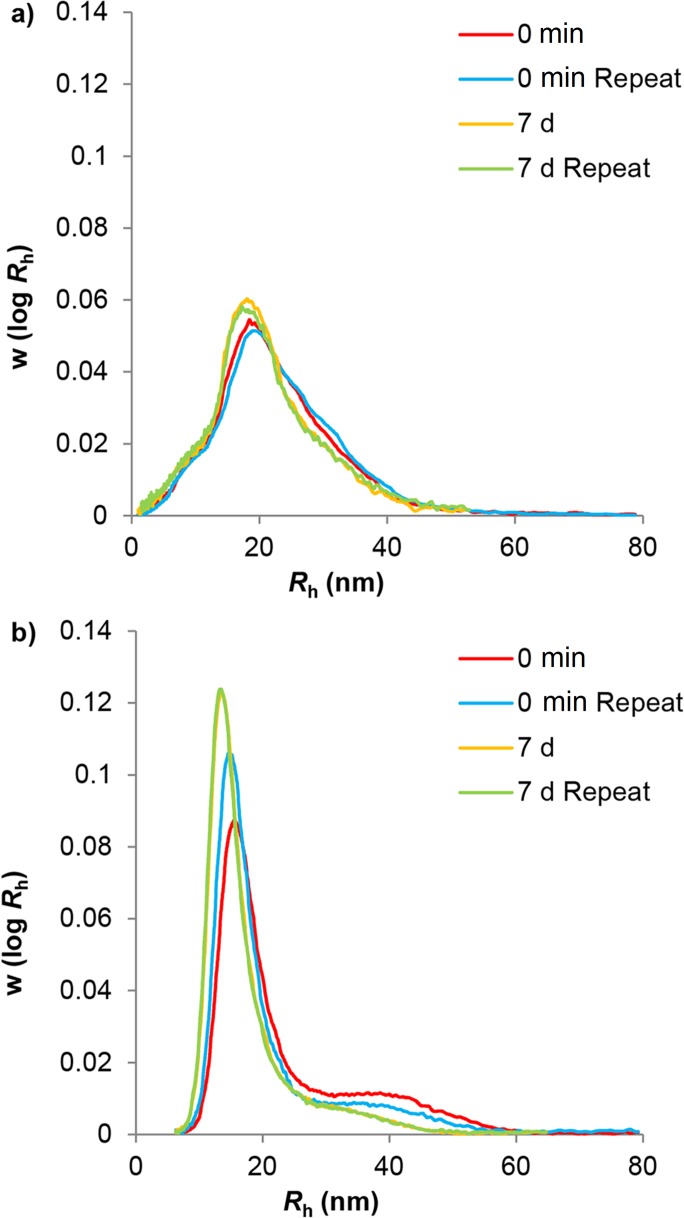
TEM images of glucans. Typical composite α particles of phytoglycogen (a) and liver glycogen (b) particles are indicated by an arrow. Scale bars are 500 nm, images taken at 50K magnification.

TEM of phytoglycogen and liver glycogen (Fig. 2) indicated the presence of α particles in glycogen and in phytoglycogen, as seen previously, e.g. [10, 11, 14, 26, 27]. These particles consist of smaller β particle subunits which are joined through an unknown binding. Phytoglycogen and liver glycogen appear to have similar morphological structures under TEM and have particles and subunits of approximately similar size which extend over a similar size range. This could possibly indicate that they have a similar structure, but one has to be very wary of such a conclusion as will be subsequently discussed.

### Acid Hydrolysis

**Fig 3:**
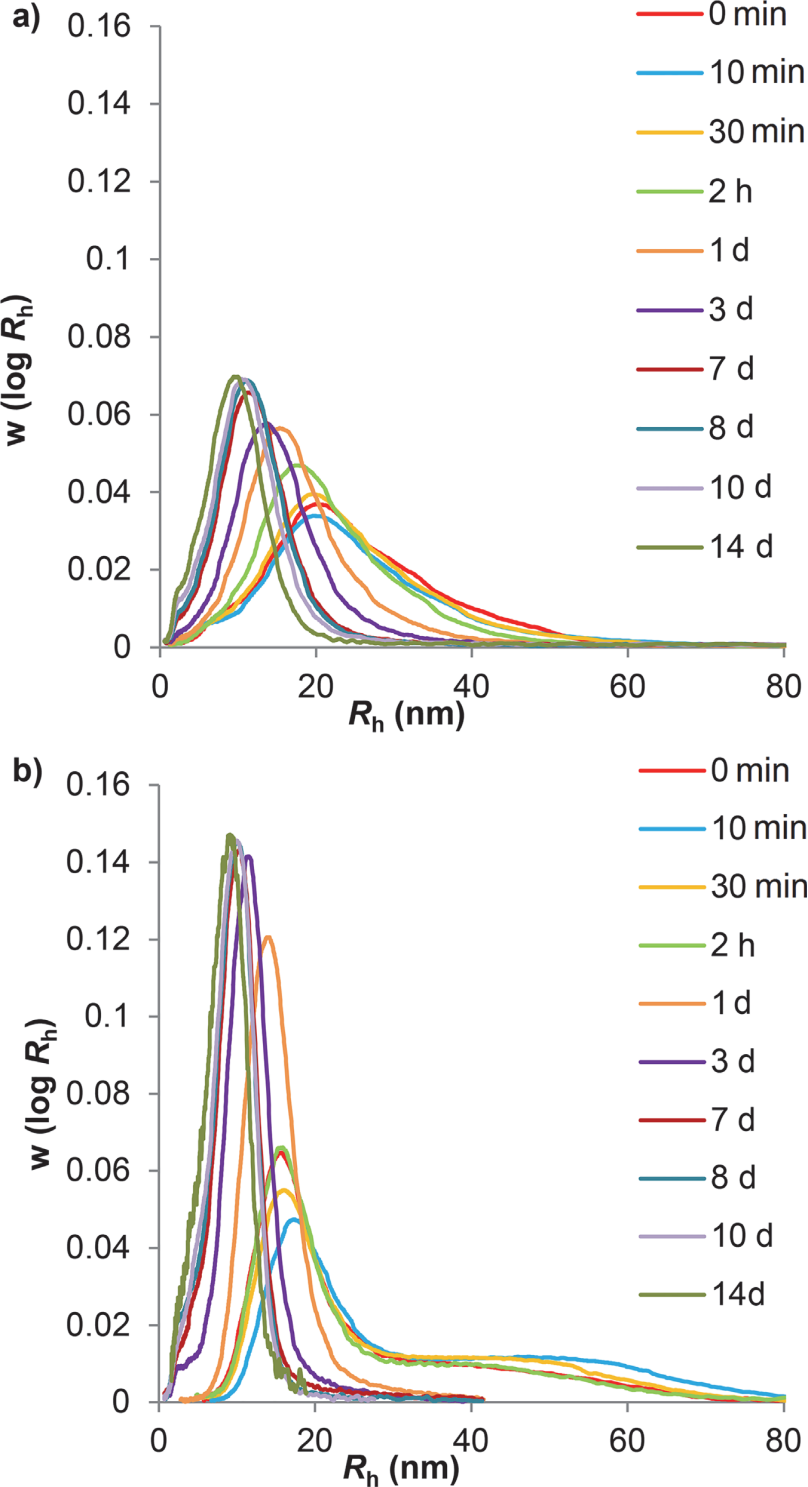
Aqueous SEC weight distributions of water-hydrolyzed glucans. Sample distributions of phytoglycogen (a) and liver glycogen (b) particles after being dissolved directly in eluent (0 min) and after heating at 80°C for 7 days (7 d). Curves have been normalized to equal areas.

Fig. 3 illustrates the effect on the size distributions of heating phytoglycogen and liver glycogen at 80°C in water. Water hydrolysis acted as a control to ensure any effects of hydrolysis is acid were not due to water hydrolysis. Phytoglycogen and liver glycogen show significantly different distributions: monomodal and bimodal respectively, the latter showing two distinct α and β particle populations. The particles for each type of glucan had a similar size range. These differences in the distributions of phytoglycogen and liver glycogen have only recently been observed, as the SEC method previously used (employing DMSO as the eluent) is unable to properly separate α- and β-particle peaks, masking this bimodality [9].

After one week of heating phytoglycogen in water, it was observed that there was only slight degradation, resulting in a small change in the overall shape of the distribution. For liver glycogen, in contrast, the larger α particle population was more hydrolyzed by water at an elevated temperature than the β particle population.

Aqueous SEC was used to characterize and analyze phytoglycogen and liver glycogen during acid hydrolysis (pH ~ 3.5) at 80°C for 10 min, 30 min, 2 h, 1 day, 3 days, 7 days, 8 days, 10 days and 14 days to observe any changes in the particle distribution (Fig. 1). This shows that liver glycogen changes from a bimodal to a monomodal distribution. The composite particles of the liver glycogen are degraded preferentially to small particles: the large composite particles in liver glycogen were completely degraded after 1 day, but the non-composite particle population was much more resistant, as previously reported [14].

While it is tempting to infer from these results that the larger component in liver glycogen is structurally different to the smaller one, this is not unambiguous. This is because one expects that acid hydrolysis will occur essentially uniformly throughout the particles, and thus larger particles will always be degraded more quickly. It is for this reason that the quantitative model of this degradation explicitly includes this size dependence (Eq. 3). The mathematical model is then used to fit the observed time evolution of the size distribution during hydrolysis, to eliminate this ambiguity in data interpretation.

### Modeling Glucan Hydrolysis

**Fig 4:**
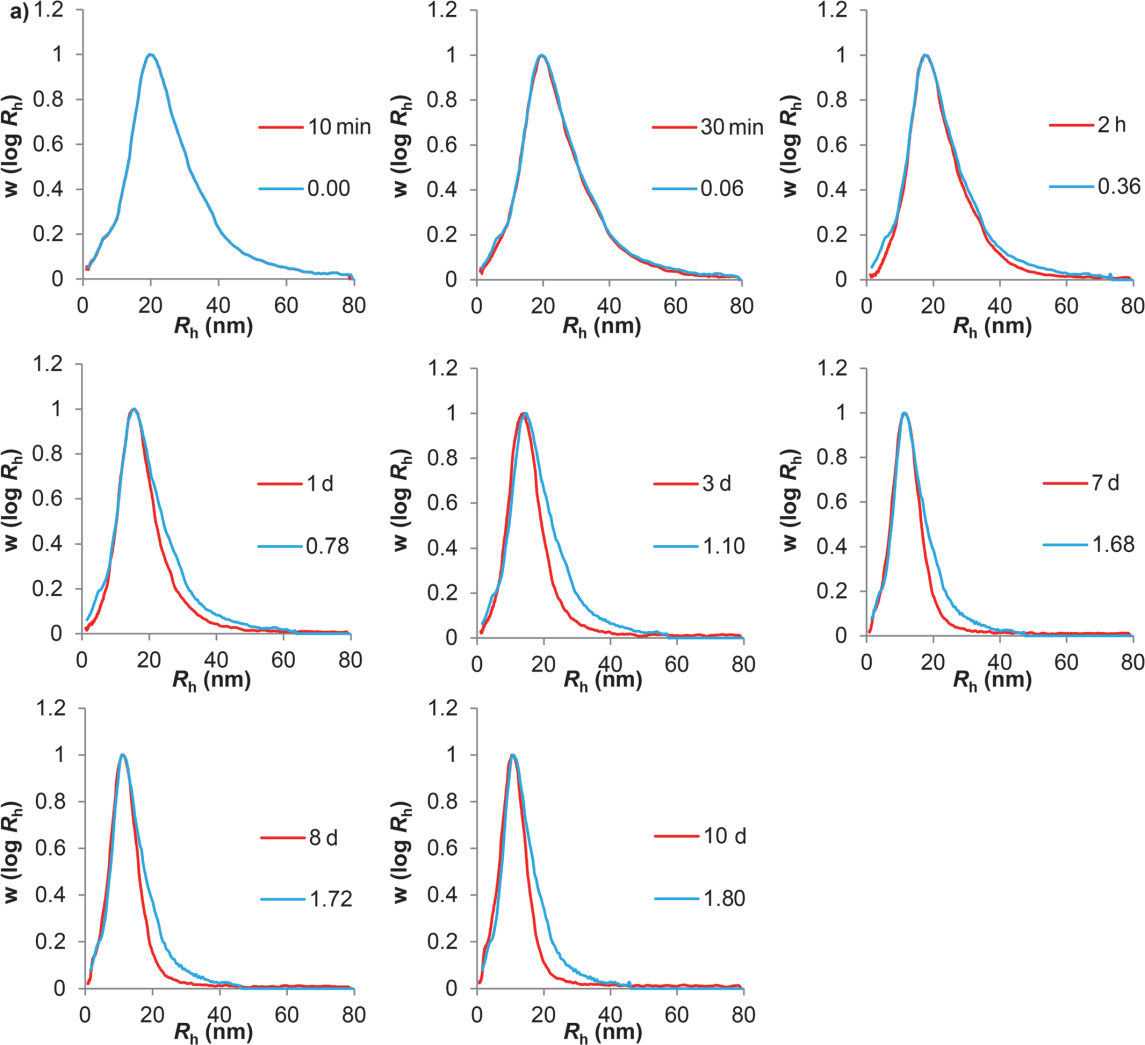
Fittings of phytoglycogen acid hydrolysis experimental data to the model of uniform degradation. Blue lines are values of the dimensionless time τ = *k/t* (see S1 Text), while red lines are experimental acid hydrolysis data. The following terms have been abbreviated: minute: min; hours: h; days: d.

**Fig 5:**
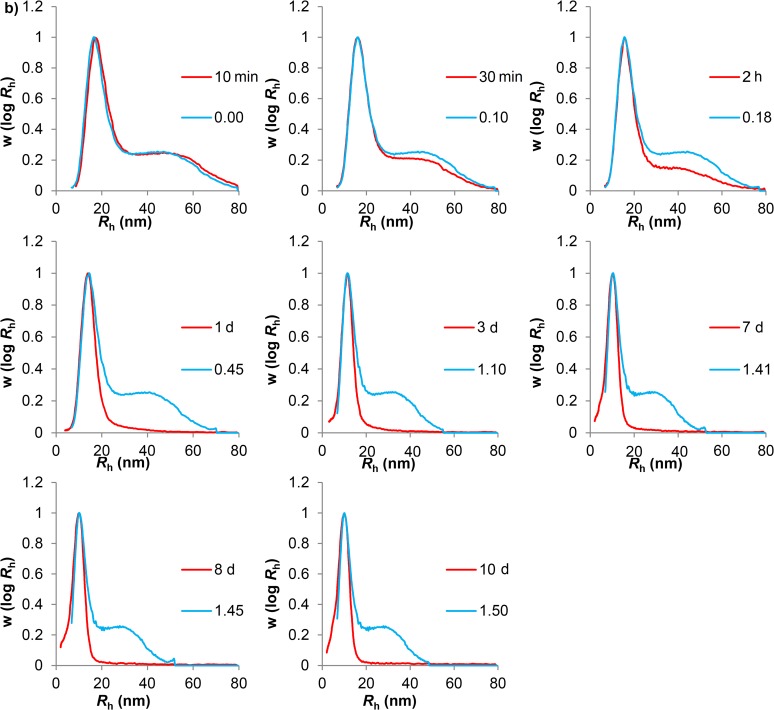
Fittings of liver glycogen acid hydrolysis data to the model of uniform degradation. Blue lines are values of the dimensionless time τ = *kt* (see S1 Text), while the red lines are the experimental acid hydrolysis data. The following terms have been abbreviated: minute: min; hours: h; days: d.

The mathematical model for the uniform degradation where non-preferential bond cleavage of glucans occurs over time was fitted to the observed time evolution (see S1 Text) of the size distribution during hydrolysis (Fig. 4 and Fig. 5). If the bonding is non-uniform (as is suspected to be the case in liver glycogen, e.g. with a covalent or strong non-covalent linkage involving a protein between glycosydically-bound β particles) then this model should fit the degradation of β but not α particles. As stated, the size distribution of each time point was calculated from inputting an initial size distribution (e.g. the 10 min data as the initial distribution for calculating 30 min, etc.). The only fitting parameter is the dimensionless time, τ = *kt*. The value of τ is chosen to give the best fit to the size distribution at the succeeding time point *t*, and the value of *k* then found as *k* = τ/*t*.

As seen in Fig. 4, the model for uniform degradation gives a relatively good fit to the shape of the phytoglycogen size distribution at each time point, although the value of *k* shows significant variation at different times. The liver glycogen data, in contrast, rapidly deviates from the model predictions. Although the model predicts that the larger component in glycogen degrades more quickly than the smaller component, the model also predicts that bimodality would be maintained, with the ratio of height of both peaks remaining constant. As can be observed in Fig. 5 and Fig. 1, the bimodality of the distribution disappeared after one day of hydrolysis. This discrepancy between model and experiment cannot be ascribed to an experimental artifact, particularly band-broadening, as proven in S1 Text and S1 Fig.

### Particle Molecular Density

As discussed in detail later, the smallest (largely β) particles, having little composite nature, would be expected to show a higher molecular density than the larger particles, which have an extensive composite nature and thus more “empty space” between the components. Thus the naïve expectation is that molecular density would be a decreasing function of molecular size, until flattening out to a final size-independent value which would be that of a sufficiently large α particle. As will be seen, the actual behavior is not this simple.

**Fig 6:**
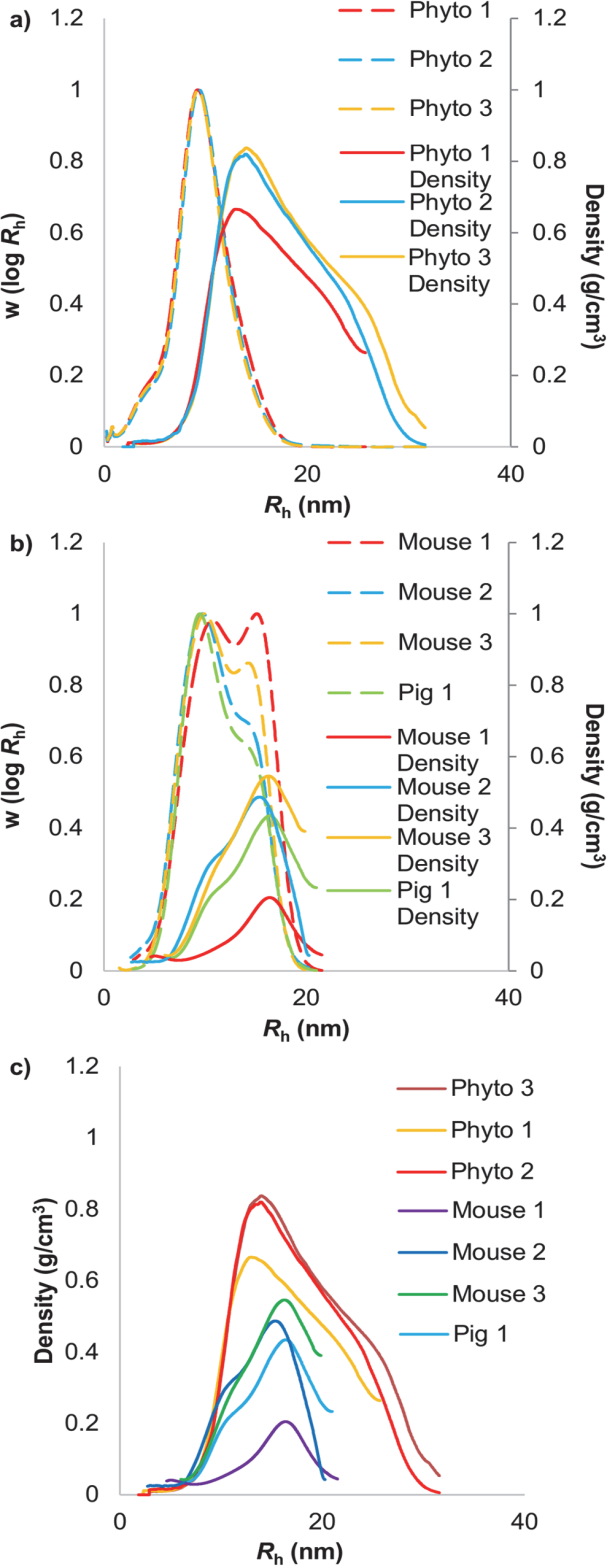
Aqueous SEC weight and molecular density distributions of glucans. Phytoglycogen (a) and liver glycogen (b) weight distributions (dashed lines) and molecular density distributions (c) (solid lines). Weight distributions have been normalized to the distribution peak while the density distributions are absolute.

Comparison of phytoglycogen and liver glycogen particle densities (those given here show more detail but are similar to those reported elsewhere [25, 27–30]) shows significant differences and similarities between the particles (Fig. 6). As expected, the densities are ~ 1 g cm–3 but slightly less than that of starch in DMSO, density ~ 1.2 g cm–3 [30]. It is seen that the density distribution of phytoglycogen shows an increase associated with the smaller particles followed by a decrease at larger sizes (extensive α particles). The densities of the phytoglycogen particles are larger within the β-particle population (small particles with little composite nature), and are significantly more dense than β particles in liver glycogen. Liver glycogen has a bimodal density distribution, where there is an initial shoulder followed by a large peak in the α-particle population before the density decreases sharply. The different size dependences and values of the densities of phytoglycogen and liver glycogen suggest that these particles differ structurally.

## Discussion

**Fig 7:**
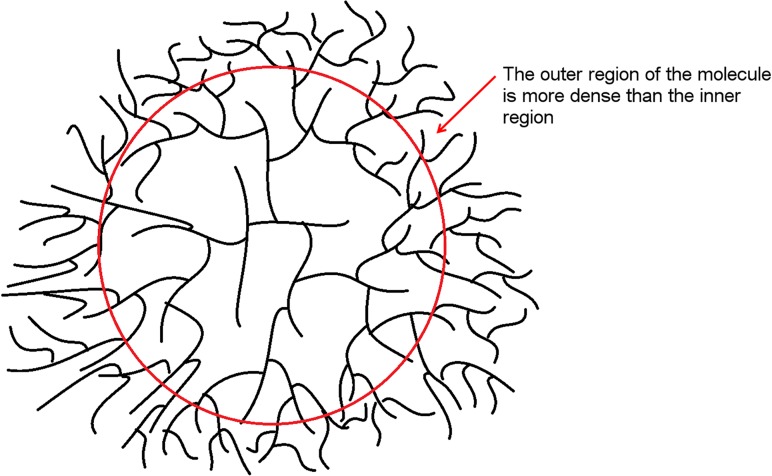
The “crowding” model of particle synthesis. The small β particle grows via the synthesis of glycosidic linkages until the density of branches in the periphery becomes so high the biosynthetic enzymes cannot operate freely, hindering growth. The dense periphery is indicated by the red circle and arrow.

There are many enzymes that both starch (and hence phytoglycogen) and glycogen utilize during their biosynthesis. Phytoglycogen and glycogen are similar in that neither have a debranching enzyme controlling branch spacing during their synthesis. This lack of debranching activity means that the glucans are unable to crystallize like normal starch [31]. Although the biosynthetic enzymes for the glycosidic bonds in glycogen and phytoglycogen are similar, they are not the same. This suggests that a major determinant of the size of β particles (which are very similar for both polymers) is physical rather than biochemical. This is consistent with mathematical models and is termed a “crowding” mechanism [14, 27, 32–37]. In this mechanism, as a β particle grows through addition of anhydroglucose monomer units, the local molecular density in the periphery eventually becomes so high that the biosynthetic enzymes for this process cannot operate freely, hindering further growth (Fig. 7). This “crowding” mechanism is absent in amylopectin, where the branching structure (especially the close spacing between branches) is quite different from that in (phyto)glycogen; this is the reason that starch can crystallize while (phyto)glycogen cannot.

Synthesis of large α particles, apparently similar to those observed in Fig. 2, has been achieved by incubating glucose-1-phosphate with phosphorylase and branching enzyme in an *in vitro* system [38]. As a glucan morphologically resembling phytoglycogen is formed in this simple *in vitro* system [38], it is unlikely that phytoglycogen is synthesized via any enzyme that would involve enzymatic action outside of normal starch biosynthesis. This suggests that the bonds used to form large composite particles in phytoglycogen are glycosidic, and indicates that phytoglycogen is most probably formed via the “crowding” mechanism previously outlined.

**Fig 8:**
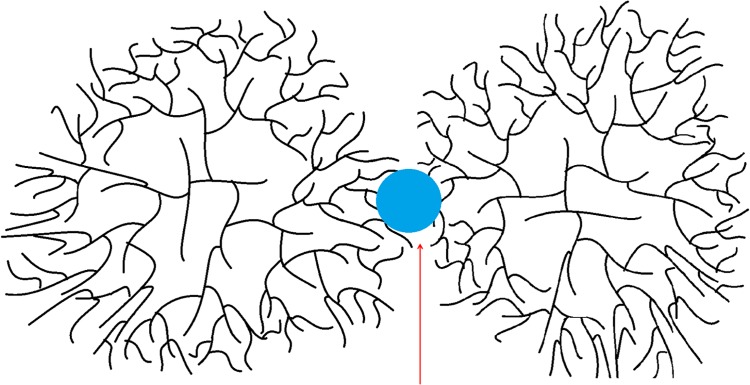
The “crowding-assembly” model of particle synthesis. The small β particles are linked (red arrow) to form larger alpha particles

As was observed in Fig. 1 and Fig. 3, the large α particle population of liver glycogen was hydrolyzed faster than the similar sized population of phytoglycogen, suggesting a different binding mechanism. This is consistent with similar work hypothesizing that peptides may be the bonding factor which results in the formation of large α particles in liver glycogen [14]. Peptide hydrolysis is readily catalyzed by acidic conditions. Additionally, there are many more proteins present in the liver in addition to those involved in glycogen biosynthesis [8] which may have a possible role as a bonding factor. The extraction methods used here have been shown to also extract contaminating proteins; however, due to the much smaller size of proteins compared to glycogen, there is no overlap of the protein contamination peak with the glycogen peak in the SEC weight distributions [39]. Furthermore, any protein which is strongly linked to the glycogen is inherently part of the molecule and thus of its characterization. Complete hydrolysis of proteins requires low pH values and high temperatures, while partial hydrolysis of proteins can occur under much milder conditions and can occur spontaneously in water at neutral pH [40, 41]. Because a protein backbone is covalently linear, the hydrolysis of a single peptide bond would be enough to sever any link involving a protein (Fig. 8) [14]. In addition, it has been previously shown that the rate of hydrolytic degradation of liver-glycogen α particles is more consistent with protein rather than glycosidic-linkage degradation.

An alternative hypothesis is that multiple glycosidic linkages bond β particles together. This is similar to the hypothesized mechanism for the formation of phytoglycogen particles; however, in liver glycogen it is likely that if glycosidic linkages are responsible for the formation of composite particles, there are fewer linkages present than in similar sized phytoglycogen particles. This is because the large liver glycogen particles are more acid-labile than their phytoglycogen counterparts.

By fitting the results of uniform acid hydrolysis of the glucans (as seen in Fig. 4 and Fig. 5) to the distributions generated by a model that assumes uniform degradation (as observed in S1 Fig.), two important observations were made: a) phytoglycogen fits the uniform-degradation model well as far as size distribution is concerned, although the rate coefficient *k* varies with the extent of degradation; and b) liver glycogen rapidly and qualitatively deviates from the predicted distribution: in particular, the uniform-degradation model predicts that the α-particle population would remain present. The disappearance of the α-particle population of liver glycogen can be most easily explained if the dominant mode of breakdown for liver glycogen is directly into smaller β particles. The degradation of phytoglycogen, on the other hand, appears to be consistent with non-preferential bond cleavage throughout all regions of the molecule. The relatively good fit of the β particle population of liver glycogen to the model is consistent with non-preferential bond cleavage and thus indicates β particles are likely to link through glycosidic linkages, as has been demonstrated in the past [36]. These results demonstrate conclusively that the bonding leading to the formation of α particles in liver glycogen is different from that leading to the formation of β particles.

**Fig 9:**
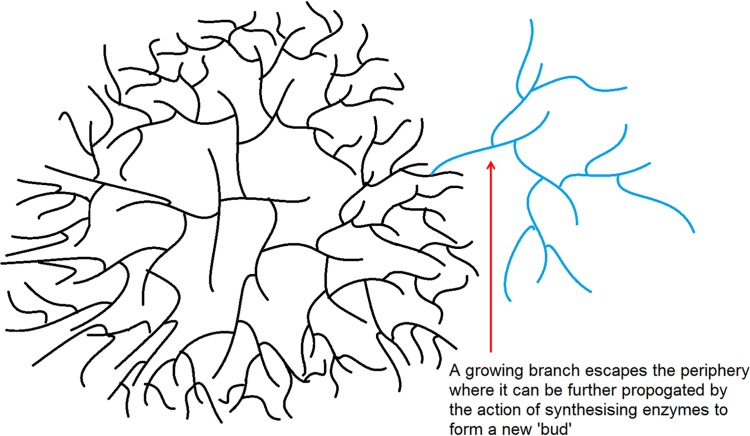
The “crowding/budding” model of particle synthesis. Much like the “crowding model, however a branch is able to escape the dense periphery (red arrow) and is subsequently acted upon by biosynthetic enzymes resulting in a new ‘bud’ to form large “α-like” particles.

The uniform degradation of the particles in phytoglycogen supports the theory of a “crowding/budding” mechanism [14] which builds on the theory of “crowding” discussed previously. In a “crowding/budding” mechanism, once the maximum periphery density has been reached, budding can occur: a growing branch wanders outside the dense outer layer to the uncrowded regions outside the particle’s periphery, whereupon rapid growth can occur and a new particle can form (Fig. 9).

The density distribution of phytoglycogen can be explained by the “crowding-budding” model assuming glycosidic linkages. This model is similar to the “crowding” model described previously With an increase in branching, a theoretical maximum density is approached, leaving budding of individual branches as the only expansion mechanism, which in turn requires the branching process to start once more. Therefore, while the new bud is incomplete, the density of the molecule is less than that of a phytoglycogen molecule with no budding at a maximum density. As the probability of new buds increases with an increasing radius, the competition for space between the buds would increase, possibly resulting in impeded bud formation, whereby a bud is unable to reach maximum density. As the size of the molecule increases, the chance of impeded and incomplete buds also increases, resulting in a decrease of the density.

Liver glycogen density also increases with size initially due to the growing size of the β particle population, as outlined above. Under the “crowding-assembly” model (Fig. 8) where β particles bind together into α particles, the formation of α particles will also result in a decrease in density, as observed in the liver glycogen density distribution.

While the nature of the linkage between particles in particles in glycogen is unclear, the results obtained here and those described elsewhere are consistent with this being a covalent or strong non-covalent linkage involving a protein. Some candidates are glycogenin or a lectin.

It has been hypothesized that glycogenin is the protein responsible for the binding of β particles to form α particles, as it is the precursor for glycogen synthesis [42, 43]. Glycogenin is relatively abundant in rat and mouse livers and it has been theorized that it is possibly present on the surface of glycogen particles [8]. If there were an increase in the population of β particles, after treating the samples with protease, this would provide strong evidence that protein is responsible for the bonds to form α particles. However, there is little effect of protease (which hydrolyses amide linkages in protein) on the distributions of either phytoglycogen or liver glycogen [14], which is ascribed to the large protease molecule being hindered from diffusing within the liver glycogen molecules and thus unable to digest internally-located proteins [14] (see S2 Text and S4 Fig.).

The data are consistent with the inference that large, composite phytoglycogen particles are most likely formed via a crowding/budding mechanism, whereby steric hindrance in the periphery of small β particles slows their growth beyond a certain radius, and further growth occurs as a few chains protruding outside this radius themselves become the growth point for forming a new, but linked, β particle through a glycosidic linkage. The data are consistent with the hypothesis that small β particles within liver glycogen are most likely formed via a crowding mechanism, as are the small phytoglycogen “β-like” particles, and that liver-glycogen β particles are synthesized separately initially and are then joined to form large α particles, a mechanism of synthesis we term the “crowding/assembling” model.

## Conclusions

Liver glycogen, a glucose polymer which is important for blood-sugar storage in animals, comprises small β particles which are linked to form much larger α particles. The present study investigated the nature of the linkage whereby β particles join together to form α particles, by comparing certain aspects of liver glycogen structure to those of phytoglycogen. Phytoglycogen was used as a tool for comparison, as although both appear to form large composite α particles, phytoglycogen contains only glycosidic linkages. Taken together, the present data on molecular density and on the time evolution of size distributions under acid hydrolysis show that liver-glycogen α particles have a different structure to liver glycogen β particles. There is circumstantial but strong evidence that the binding of β into α particles in liver glycogen is a covalent or strong non-covalent linkage involving a protein.

Given the discovery that formation of α particles is impaired in diabetes [13], and that the formation of α particles reaches a maximum some time after most glucose storage in liver glycogen has already occurred (a process that would optimize blood-sugar control in healthy animals) [12], the discoveries in the present paper on the nature of the bonding resulting in α-particle formation have significant implications for developing new types of drug targets for diabetes.

## Supporting Information

S1 Fig The model of uniform degradation over an extended time period. Each colored line indicates a different value of dimensionless time τ = *kt* as calculated from an initial liver glycogen distribution. Curves have been normalized to the distribution peak. As can be observed, using the data obtained from liver glycogen for this model, the distribution maintains its bimodality over the course of hydrolysis. If band-broadening were qualitatively affecting the distribution, it would be expected that the peak at higher hydrodynamic size would turn into an extended tail. This is qualitatively different from what is seen experimentally, suggesting that band broadening does not affect the conclusions drawn from fitting to the model.
(TIF)

S2 Fig Comparison of fittings generated from the uniform model of hydrolysis. The uniform model of hydrolysis (h) was fitted to liver glycogen to small “non-composite” particles (a-d) and to large α particles (e-g). The α-particle population can only be fitted up to 2 h as the population has disappeared by the time the next sample was taken, at 1 day. The actual (red) and fitted dimensionless (blue) times are shown. Curves have been normalized to the population peak of interest. The following terms have been abbreviated: minute: min; hours: h; days: d.
(TIF)

S3 Fig Modified crowding budding models. The full-bud model reflects the protein mediated binding-assembly model where the structure of the molecule is most likely to be loosely randomized resulting in a decreased density. The semi-bud model represents the crowding-budding model where new buds are incomplete decreasing the density of the molecules as the buds are unable to reach maximum density.
(TIF)

S4 Fig Aqueous SEC weight distributions of glucans subjected to protease treatment. Phytoglycogen (a) and pig liver glycogen (b) were treated with protease over a range of temperatures (20–80°C). Curves have been normalized to equal areas.
(TIF)

S1 TableDensities of phytoglycogen and starch samples.Molecular weight (${\bar{M}}_{\text{w}}$) and radius of gyration (*R*gz) results were taken from the literature [44]. The densities (*ρ)* of the samples were re-analyzed to include the factor of 4/3 π to enable comparison with macroscopic densities as outlined in materials and methods (Density Distributions of Phytoglycogen and Liver Glycogen).(DOCX)

S1 Text Derivation, numerical solution and data fitting of evolution equation for size distribution during acid hydrolysis. (DOCX)

S2 Text Effects of proteases on glucan structure. (DOCX)
