# Correction: Acid Hydrolysis and Molecular Density of Phytoglycogen and Liver Glycogen Helps Understand the Bonding in Glycogen α (Composite) Particles

**DOI:** 10.1371/journal.pone.0134065

**Published:** 2015-07-24

**Authors:** Prudence O. Powell, Mitchell A. Sullivan, Joshua J. Sheehy, Benjamin L. Schulz, Frederick J. Warren, Robert G. Gilbert

Figs 1, 2, and 3 appear in the incorrect order in the published article. Please view correct versions of the figures here.

**Fig 1:**
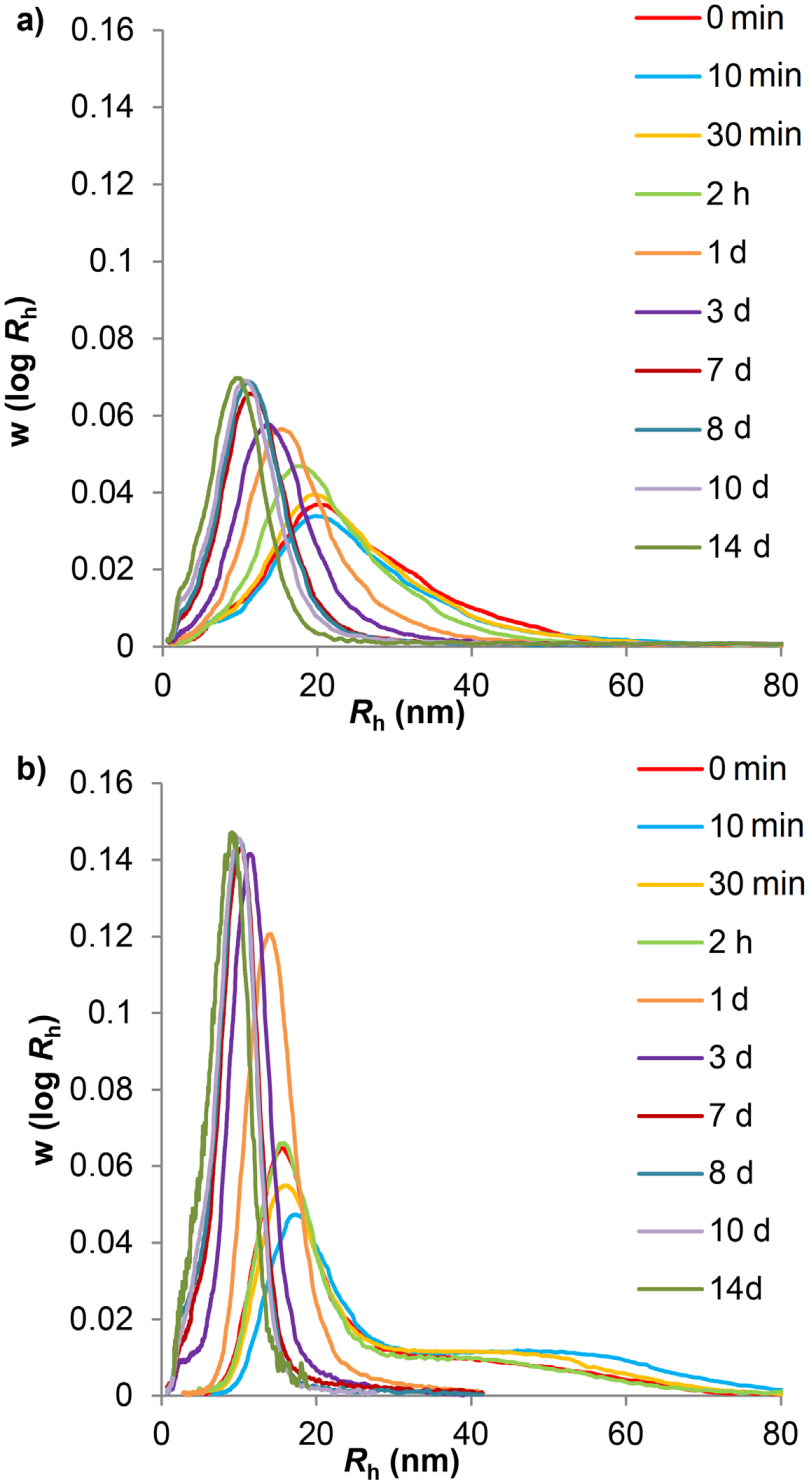
Aqueous SEC weight distributions of acid hydrolyzed glucans. Phytoglycogen (a) and liver glycogen (b) particle samples were taken over 14 days of acid hydrolysis. The following terms have been abbreviated: minute: min; hours: h; days: d. Curves have been normalized to equal areas.

**Fig 2:**
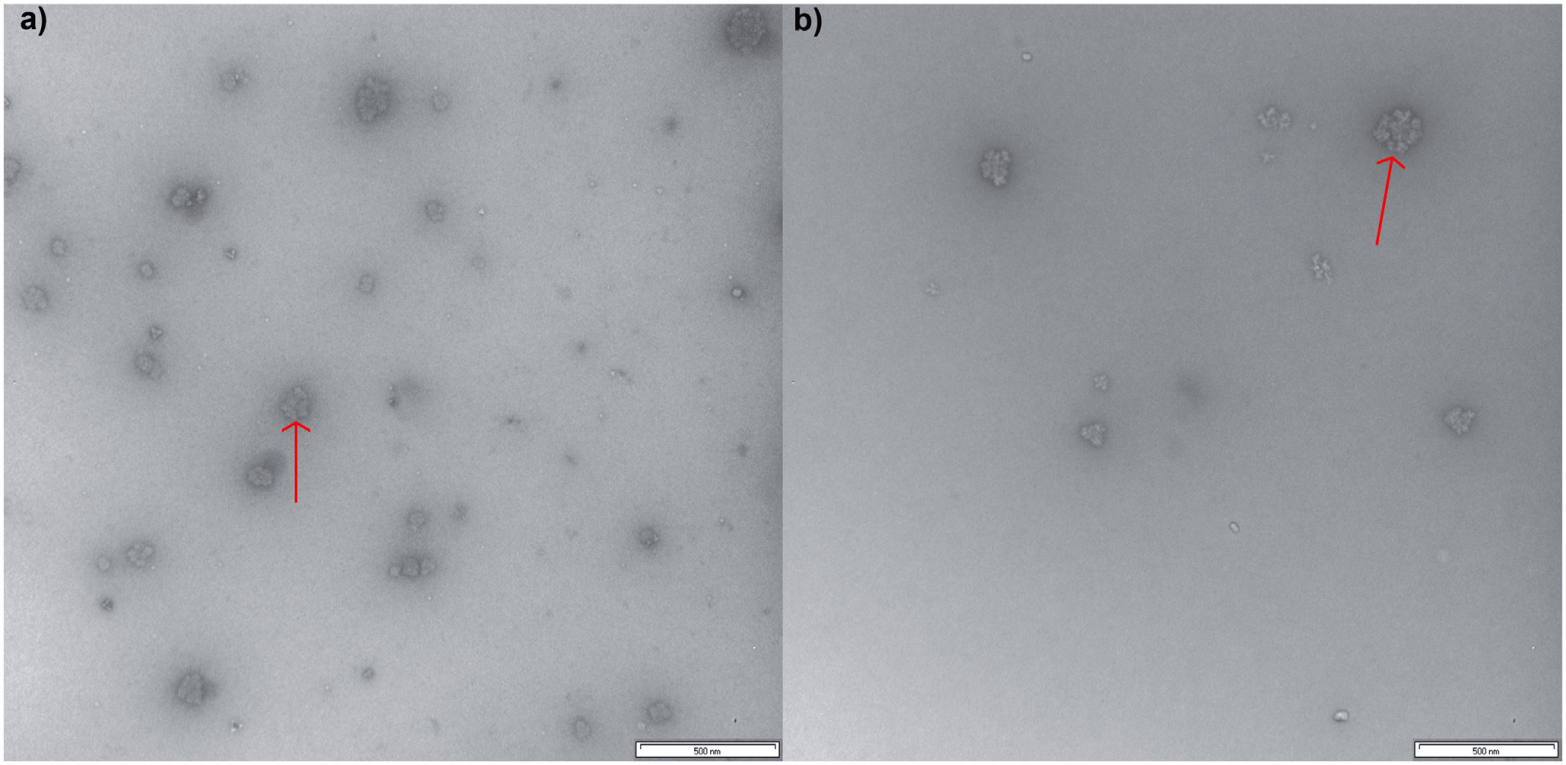
TEM images of glucans. Typical composite α particles of phytoglycogen (a) and liver glycogen (b) particles are indicated by an arrow. Scale bars are 500 nm, images taken at 50K magnification.

**Fig 3:**
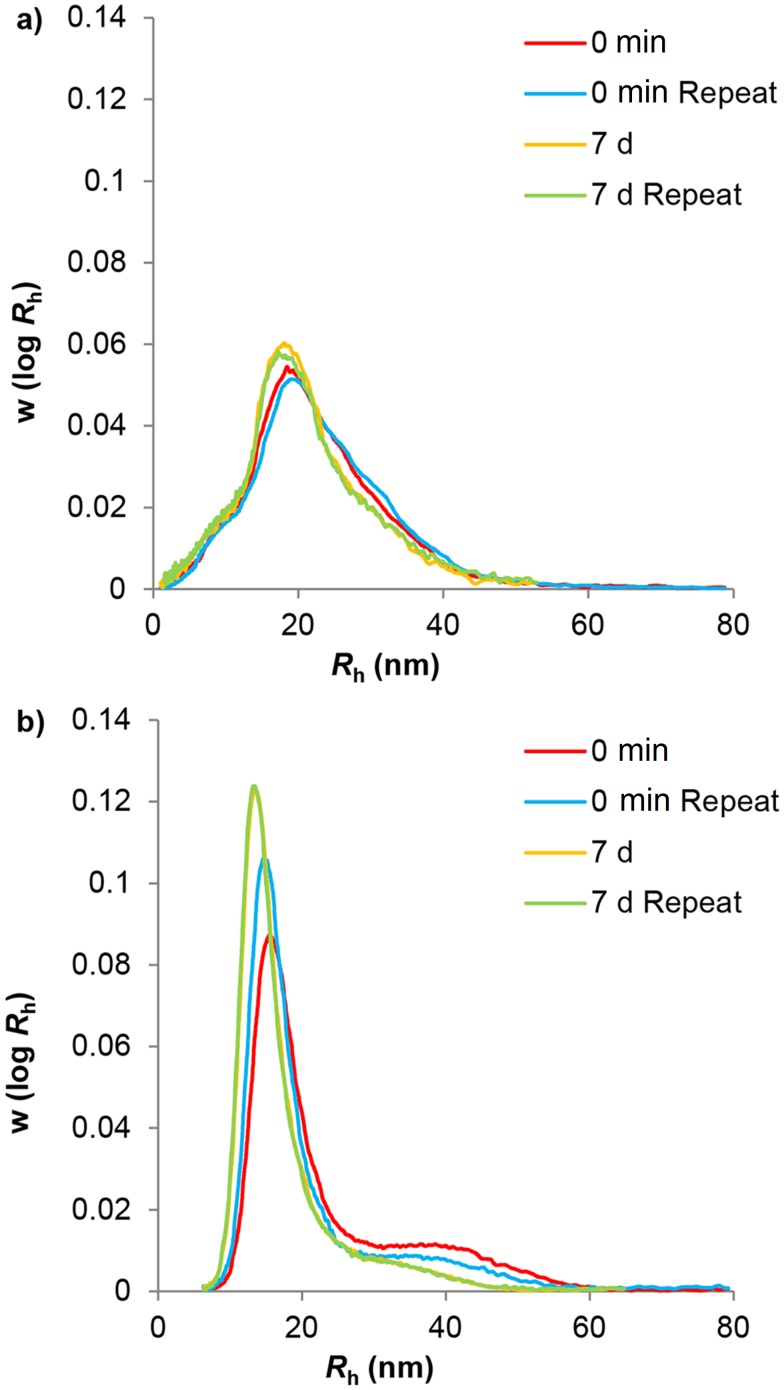
Aqueous SEC weight distributions of water-hydrolyzed glucans. Sample distributions of phytoglycogen (a) and liver glycogen (b) particles after being dissolved directly in eluent (0 min) and after heating at 80°C for 7 days (7 d). Curves have been normalized to equal areas.
