# Supplementary figures and images for: Acid Hydrolysis and Molecular Density of Phytoglycogen and Liver Glycogen Helps Understand the Bonding in Glycogen α (Composite) Particles
Source: PLoS One. 2015 Mar 23;10(3):e0121337. doi: 10.1371/journal.pone.0121337 (PMC4370380; doi:10.1371/journal.pone.0121337)

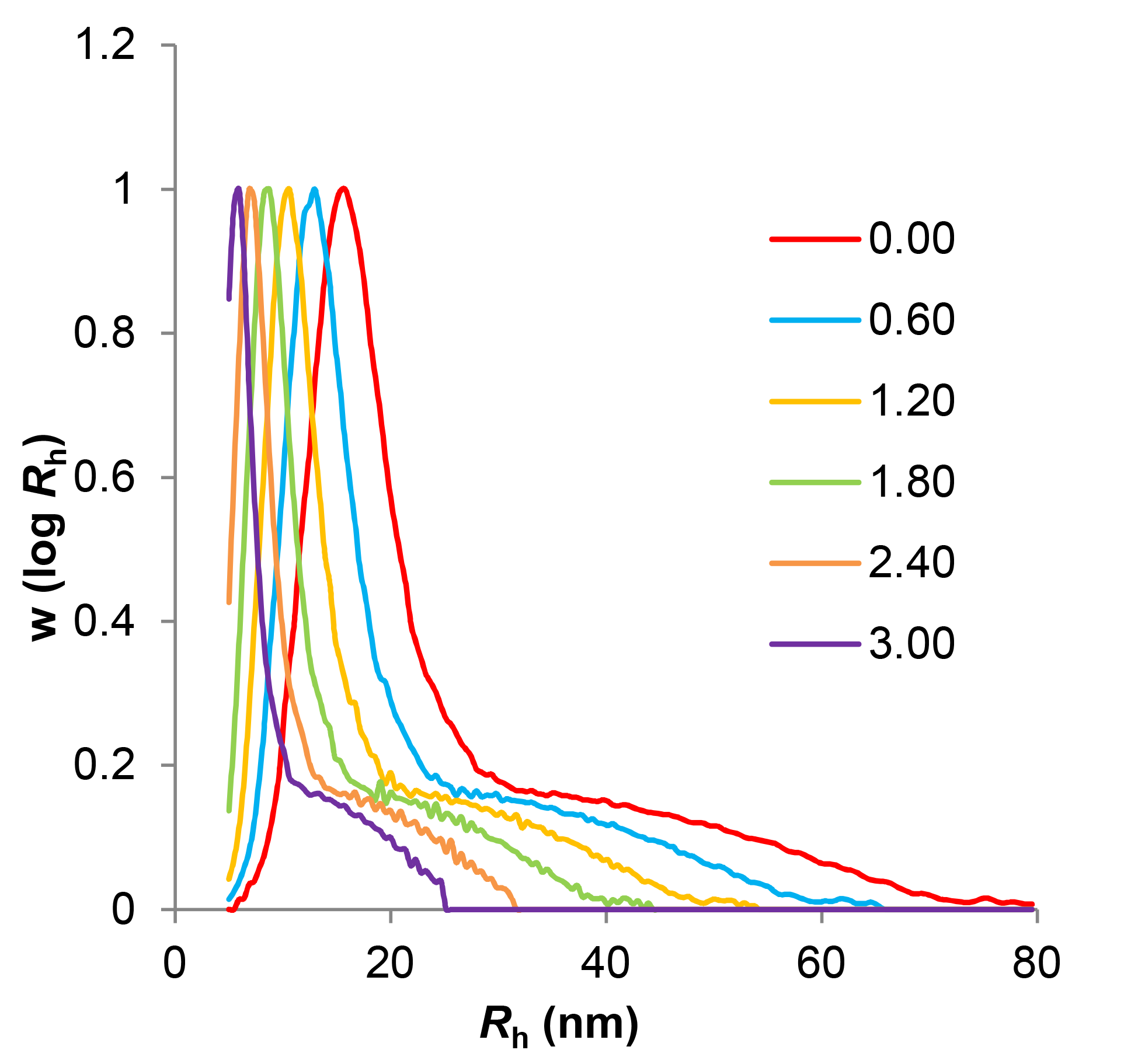

Supplement: S1 Fig — Each colored line indicates a different value of dimensionless time τ = kt as calculated from an initial liver glycogen distribution. Curves have been normalized to the distribution peak. As can be observed, using the data obtained from liver glycogen for this model, the distribution maintains its bimodality over the course of hydrolysis. If band-broadening were qualitatively affecting the distribution, it would be expected that the peak at higher hydrodynamic size would turn into an extended tail. This is qualitatively different from what is seen experimentally, suggesting that band broadening does not affect the conclusions drawn from fitting to the model. (TIF) [file pone.0121337.s001.tif]

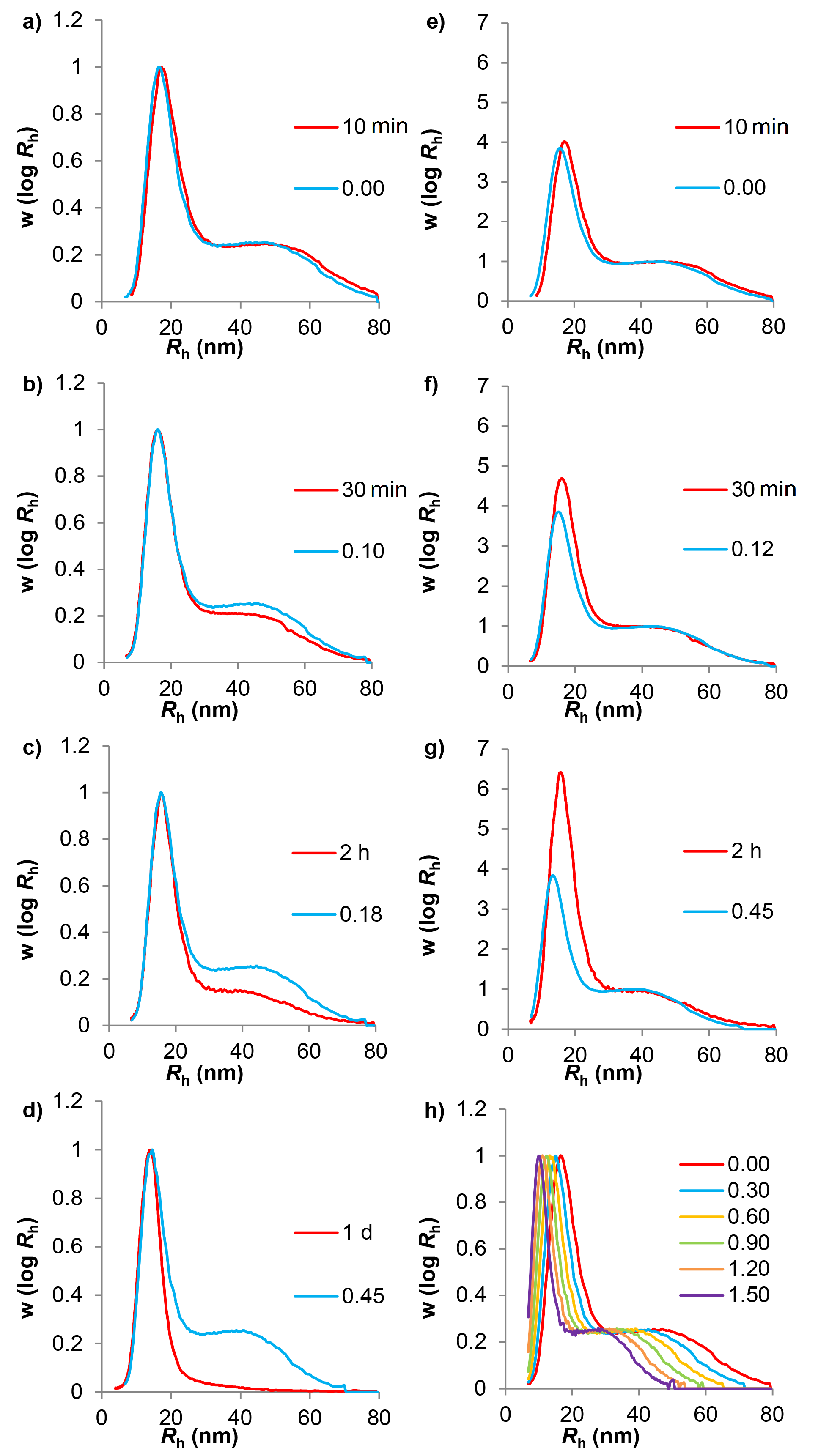

Supplement: S2 Fig — The uniform model of hydrolysis (h) was fitted to liver glycogen to small “non-composite” particles (a-d) and to large α particles (e-g). The α-particle population can only be fitted up to 2 h as the population has disappeared by the time the next sample was taken, at 1 day. The actual (red) and fitted dimensionless (blue) times are shown. Curves have been normalized to the population peak of interest. The following terms have been abbreviated: minute: min; hours: h; days: d. (TIF) [file pone.0121337.s002.tif]

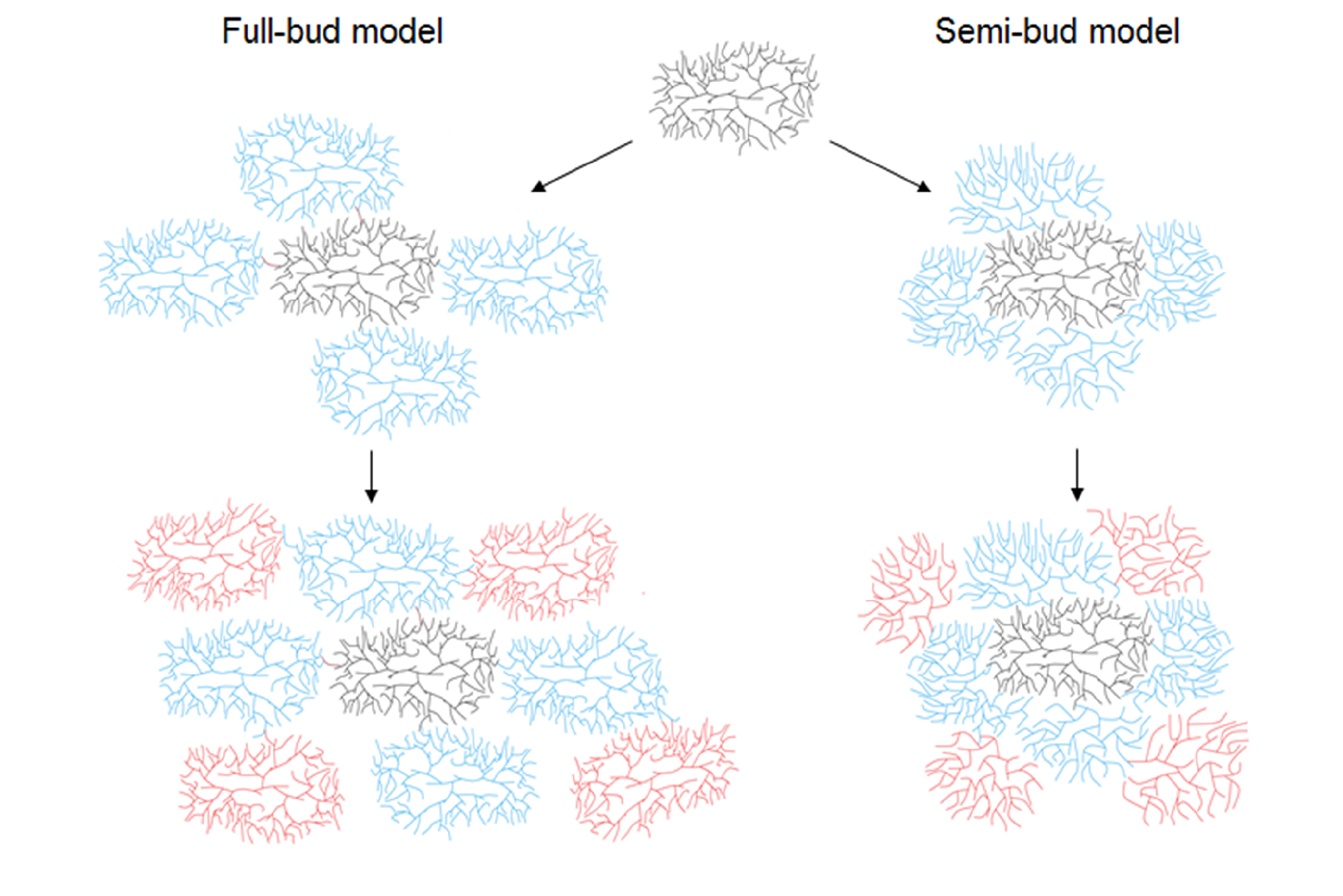

Supplement: S3 Fig — The full-bud model reflects the protein mediated binding-assembly model where the structure of the molecule is most likely to be loosely randomized resulting in a decreased density. The semi-bud model represents the crowding-budding model where new buds are incomplete decreasing the density of the molecules as the buds are unable to reach maximum density. (TIF) [file pone.0121337.s003.tif]

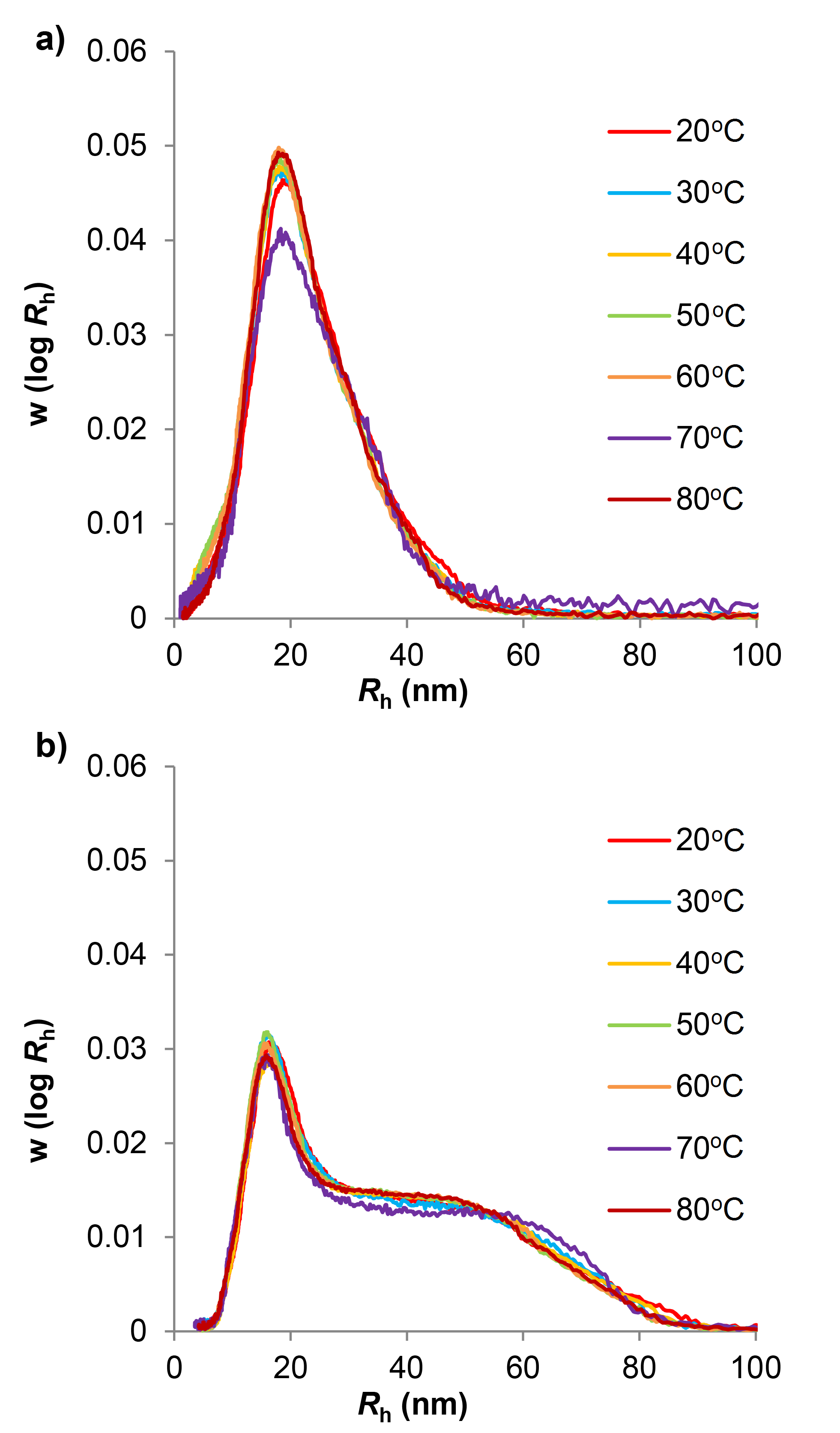

Supplement: S4 Fig — Phytoglycogen (a) and pig liver glycogen (b) were treated with protease over a range of temperatures (20–80°C). Curves have been normalized to equal areas. (TIF) [file pone.0121337.s004.tif]
