# Supplementary material for: Acid Hydrolysis and Molecular Density of Phytoglycogen and Liver Glycogen Helps Understand the Bonding in Glycogen α (Composite) Particles
Source: PLoS One. 2015 Mar 23;10(3):e0121337. doi: 10.1371/journal.pone.0121337 (PMC4370380; doi:10.1371/journal.pone.0121337)
Supplement: S1 Table — Molecular weight (M¯w) and radius of gyration (Rgz) results were taken from the literature [44]. The densities (ρ) of the samples were re-analyzed to include the factor of 4/3 π to enable comparison with macroscopic densities as outlined in materials and methods (Density Distributions of Phytoglycogen and Liver Glycogen). (DOCX) [file pone.0121337.s005.docx]

**Table S1.** **Densities of phytoglycogen and starch samples.** Molecular weight ($\overline{M}_{\text{w}}$) and radius of gyration (*R*gz) results were taken from the literature [[53](#_ENREF_53)]. The densities (*ρ)* of the samples were re-analyzed to include the factor of 4/3 π to enable comparison with macroscopic densities as outlined in materials and methods (Density Distributions of Phytoglycogen and Liver Glycogen).

|  | **Samples** | $\overline{M}_{\text{w}}$ | ***R*gz** | **ρ (g cm**–3**)** |
| --- | --- | --- | --- | --- |
| **Starch** | TC65 | 2.85 × 108 | 159.3 | 0.028 |
|  | EM653 | 1.93 × 108 | 120.4 | 0.044 |
|  | EM935 | 2.04 × 108 | 121.7 | 0.045 |
|  | EM937 | 2.50 × 108 | 95.4 | 0.114 |
| **Phytoglycogen** | EM653 | 6.90 × 107 | 39.9 | 0.431 |
|  |  | 2.10 × 107 | 27.7 | 0.392 |
|  |  | 1.40 × 107 | 23.1 | 0.450 |
|  |  | 8.00 × 10 6 | 15.3 | 0.886 |
|  | EM935 | 5.80 × 107 | 37.1 | 0.450 |
|  |  | 2.00 × 107 | 25.4 | 0.484 |
|  |  | 1.40 × 107 | 20.6 | 0.635 |
|  |  | 9.00 × 106 | 14.7 | 0.635 |
|  | EM937 | 5.00 × 107 | 35.6 | 1.124 |
|  |  | 2.10 × 107 | 24.1 | 0.439 |
|  |  | 1.50 × 107 | 20.9 | 0.595 |
|  |  | 1.00 × 107 | 15.6 | 0.652 |
|  | EM914 | 4.50 × 107 | 42.7 | 1.045 |
|  |  | 1.90 × 107 | 31.8 | 0.229 |
|  |  | 1.40 × 107 | 21.5 | 0.234 |
