# Supplementary material for: Acid Hydrolysis and Molecular Density of Phytoglycogen and Liver Glycogen Helps Understand the Bonding in Glycogen α (Composite) Particles
Source: PLoS One. 2015 Mar 23;10(3):e0121337. doi: 10.1371/journal.pone.0121337 (PMC4370380; doi:10.1371/journal.pone.0121337)
Supplement: S1 Text — (DOCX) [file pone.0121337.s006.docx]

## Text S1. Derivation, numerical solution and data fitting of evolution equation for size distribution during acid hydrolysis.

Consider the time evolution of evolution for a volume increment of glycogen particles under uniform degradation. An example of the model for the uniform degradation of liver glycogen over an extended time period can be seen in S1.

Break the volume into small sections labeled *i* separated by equal increments ∆*V*. Uniform degradation means that there is a loss in box *i* caused by degradation into box *i* –1, and a gain caused by degradation from box *i* + 1. The number per unit time is inversely proportional to ∆*V*. Thus the rate equation for box *i* is:

$\frac{dN_{i}}{dt}\text{ = K }\frac{N_{i+1}N_{i}}{\Delta V}$ (1)

On taking the limit as the box size ∆*V* approaches zero, the second term on the right-hand side becomes the derivative ∂*N*/∂*V*. One then has:

$\frac{\partial N(R,t)}{\partial t}\text{ = }\frac{\partial}{\partial V}(KN)$ (2)

If this rate is uniform and random, then one has

$K\left(V\right)=kV$ (3)

where *k* is a constant. One then transforms variables in eq 2 from *N* to *w* using the relation $w\left(\text{log}R\right)=\left[M\left(R\right)\right]^{2 }N(R)$, and from *V* to *R*h. This change of variables is because both *N* and *V* vary over many orders of magnitude in the rages of each where there is significant mass of polymer. This can cause difficulties with numerical solutions. However, both *w* and *R*h vary by relatively small amounts over the range of significant mass of polymer. The result is the following first-order partial differential equation:

$\frac{\partial w(R,t)}{\partial t}\text{ = k }\left(\frac{1}{3}R\frac{\partial w}{\partial R}-w\right)$ (4)

This is solved numerically by finite difference. Chose evenly spaced division of the range of *R* with increment ∆*R*. The lowest value of *R* is denoted *R*min. At time *t*, the *i*th element of the array *wi* is *w*(*R*), with

$i\text{=1+}\frac{R-R_{min}}{\Delta R}$ (5)

The derivative ∂*w/*∂*R* is expressed in terms of finite difference. Usually it is sufficient to use a second-order expression for this, but it was found that for the particular case considered here, a fourth-order centered-difference expression was needed for numerical stability:

$\partial w\text{/}\partial R\to\frac{\text{d}w_{i}}{\text{d}R_{i}}\text{ = }\frac{1}{12\Delta R}\text{ (}-w_{i+2}\text{+8}w_{i+1}-8w_{i-1}\text{+}w_{i-2}\text{)}$ (6)

with *R* and *i* related by eq 5. The first and last two terms use forward and backward differences respectively, with all the rest using the centered difference. The forward and backward difference terms are, respectively:

$\partial w\text{/}\partial R\to\frac{\text{d}w_{i}}{\text{d}R_{i}}\text{ = }\frac{1}{2\Delta R}\text{ (}-w_{i+2}\text{+4}w_{i+1}-3w_{i}\text{), }\frac{1}{2\Delta R}\text{ (}w_{i+2}-4w_{i+1}\text{+}3w_{i}\text{)}$ (7)

If one changes the time variable from *t* to the dimensionless quantity τ = *kt*, then the solution has no adjustable parameters. Thus if one chooses the value of τ so that the size distribution gives the best match to experiment, and that is at an actual time *t*, then the value of *k* is given by *k* = τ / *t*. This gives a fitted value of *k*. Liver glycogen was fitted to both α and β particle peaks (S2).
