# Supplementary material for: Acid Hydrolysis and Molecular Density of Phytoglycogen and Liver Glycogen Helps Understand the Bonding in Glycogen α (Composite) Particles
Source: PLoS One. 2015 Mar 23;10(3):e0121337. doi: 10.1371/journal.pone.0121337 (PMC4370380; doi:10.1371/journal.pone.0121337)
Supplement: S2 Text — (DOCX) [file pone.0121337.s007.docx]

## Text S3. Effects of Proteases on Glucan Structure

Phytoglycogen and pig glycogen (14 mg each) were added to protease type XIV from *Streptomyces grisens* (2.5 units/mL; P5 147-1G) in tricine buffer (3.5 mL; pH 7.5, 250 mM). The solution (0.5 mL) was dispensed into Eppendorf^®^ tubes and placed in a thermomixer for 30 min at 20 °C. Every 30 min a tube of each sample was removed and the temperature increased by 10 °C until a temperature of 80 °C was reached. The removed samples were precipitated with four volumes of absolute ethanol and centrifuged (4000 *g*, 10 min). Samples were then dissolved in the DMSO/LiBr (0.5 % w/w) eluent and characterized by SEC. The same experiment was perfomed exactly as above, but with samples dissolved in aqueous (ammonium nitrate) eluent for aqueous SEC rather than DMSO/LiBr (0.5 % w/w). All samples were prepared in duplicate.

To test if any protein was associated with the bimodal distribution of liver glycogen that may be a factor in the formation of composite particles, samples were treated with protease. S3 demonstrates the effect of protease at varying temperatures on the size distributions of phytoglycogen and liver glycogen. It was observed previously that protease had little effect on the size of liver glycogen at 37 °C [[1](#_ENREF_1)] and it was hypothesized that steric hindrance prevented the protease from diffusing within the molecules. Therefore, a range of temperatures were tested in this study as it was theorized that heating phytoglycogen and liver glycogen may result in the expansion of the molecules thus allowing protease to diffuse within the interior of the molecule and digest any internally located proteins that may aid in the creation of composite particles. It was seen, however, that protease treatment does not have a significant effect on the size of any of the particles, consistent with past experiments [[1](#_ENREF_1)].

[1] Sullivan MA, O’Connor MJ, Umana F, Roura E, Jack K, et al. (2012) Molecular Insights into Glycogen Alpha-Particle Formation. Biomacromolecules 13: 3805-3813.
